# Safety, tolerability, and acceptability of long-acting injectable cabotegravir for HIV prevention in cisgender female adolescents (HPTN 084-01): a single-arm, open-label, phase 2b trial

**DOI:** 10.1016/S2352-3018(24)00310-2

**Published:** 2025-03-12

**Authors:** Lynda Stranix-Chibanda, erica l hamilton, Julie Ngo, Yuqing Jiao, Brett Hanscom, Rahul Paul Choudhury, Yaw Agyei, Estelle Piwowar-Manning, Mark Marzinke, Sinead Delany-Moretlwe, Nyaradzo Mgodi, Bekezela Siziba, Ishana Naidoo, Brenda Gati Mirembe, Betty Kamira, Cynthia McCoig, Adeola Adeyeye, Hans M L Spiegel, Sybil Hosek

**Affiliations:** a University of Zimbabwe Clinical Trials Research Centre, Harare, Zimbabwe; b Faculty of Medicine and Health Sciences, University of Zimbabwe, Harare, Zimbabwe; c Network and Collaborative Research Division, FHI 360, Durham, NC, USA; d Statistical Center for HIV/AIDS Research and Prevention (SCHARP), Fred Hutchinson Cancer Research Center, Seattle, WA, USA; e HPTN Laboratory Center, Johns Hopkins University, Baltimore, MD, USA; f Wits Reproductive Health and HIV Institute, University of the Witwatersrand, Johannesburg, South Africa; g MU-JHU Research Collaboration, Kampala, Uganda; h ViiV Healthcare, Madrid, Spain; i National Institute of Allergy and Infectious Diseases (NIAID), Rockville, MD, USA; j Kelly Government Solutions, Rockville, MD, USA; k Department of Medicine, University of Illinois Chicago, Chicago, IL, USA

## Abstract

**Background:**

Long-acting formulations of HIV pre-exposure prophylaxis (PrEP) appear particularly well suited to adolescents. We aimed to establish the safety, tolerability, and acceptability of long-acting injectable cabotegravir as PrEP in cisgender adolescent girls.

**Methods:**

HPTN 084-01 is a single-arm, open-label, phase 2b trial conducted at three clinical research sites in South Africa, Uganda, and Zimbabwe. Girls were recruited via community study-outreach teams, reproductive health clinics, and peer referral. Sexually active adolescent girls (younger than 18 years) willing to use long-acting contraception, weighing at least 35 kg, and able to participate with parental or guardian consent (unless an emancipated minor) were eligible. After an oral lead-in, if no adverse events occurred, participants received a 3 mL intramuscular gluteal injection (long-acting injectable cabotegravir 600 mg) at weeks 5, 9, 17, 25, and 33. The product was discontinued for grade 3 or higher toxic effects or pregnancy. The primary outcomes were safety, tolerability, and acceptability. Safety (ie, proportions of grade 2 or higher clinical and laboratory events) was assessed at weeks 6, 10, 18, 26, and 34 in all enrolled participants. Injection tolerability (ie, proportions of premature discontinuation due to intolerability, frequency of injections, or burden of study procedures) and product acceptability (ie, proportions of scheduled injections completed and participants preferring long-acting injectable cabotegravir for future use) were assessed in all participants who received at least one injection at study end. The trial was registered with ClinicalTrials.gov (NCT04824131) and is completed.

**Findings:**

Between Nov 1, 2020, and Aug 31, 2021, 69 participants were assessed for eligibility and 55 met inclusion criteria. The mean age was 16·0 years (SD 1·1), 39 (71%) had a recent primary sexual partner, 12 (22%) reported transactional sex, and 22 (40%) had sexually transmitted infections at baseline. Two participants dropped out and did not initiate long-acting injectable cabotegravir due to adverse events unrelated to the study drug during the oral lead-in. One participant stopped long-acting injectable cabotegravir after three injections due to pregnancy. 51 (93%) participants reported at least one adverse event of grade 2 or higher, mostly unrelated, transient laboratory abnormalities. There were no long-acting injectable cabotegravir discontinuations due to intolerability. Of the 52 participants who completed step 2, all scheduled injections were completed and 32 (62%) participants reported they would consider using long-acting injectable cabotegravir for HIV prevention in the future.

**Interpretation:**

Long-acting injectable cabotegravir is a safe, tolerable, and acceptable option for the prevention of HIV in adolescent girls. Our study findings expand the HIV prevention options available to adolescent girls.

**Funding:**

National Institute of Allergy and Infectious Diseases, National Institute of Mental Health, National Institute on Drug Abuse, the Eunice Kennedy Shriver National Institute of Child Health and Human Development, ViiV Healthcare, and The Bill & Melinda Gates Foundation.

## Introduction

Adolescent girls and young women are a priority population for HIV prevention in eastern and southern Africa, where one in four people who acquired HIV in 2023 were adolescent girls and young women aged 15–24 years.^1^ In adult cisgender women enrolled in the HPTN 084 trial (NCT03164564), long-acting injectable cabotegravir administration was shown to be superior to daily oral tenofovir disoproxil fumarate–emtricitabine for HIV pre-exposure prophylaxis (PrEP).^2^ The safety profile and pharmacokinetics of long-acting injectable cabotegravir were acceptable for registration as a prevention option for use in adults and adolescents weighing at least 35 kg as part of a combination HIV prevention approach.^3^ The HPTN 084 study participants valued the discretion and ease of use of long-acting injectable cabotegravir, which outweighed their concerns about injections.^4^ In 2022, WHO included long-acting injectable cabotegravir in the methods recommended as HIV PrEP for cisgender women as an alternative option to daily oral tenofovir disoproxil fumarate–emtricitabine or the monthly dapivirine vaginal ring.^3^ Implementation projects in 12 countries in eastern and southern Africa are scheduled to deliver long-acting injectable cabotegravir to adolescent and young women in 2024, with initiatives in South Africa, Zambia, and Zimbabwe already underway.

**Research in context**

**Evidence before this study**

Globally, young people continue to be disproportionately affected by new HIV infections, having to overcome multiple barriers to initiate and continue effective prevention strategies. Long-acting injectable cabotegravir was shown to be safe and effective when used every 8 weeks as pre-exposure prophylaxis (PrEP) by adult men and women in the the HIV Prevention Trials Network's (HPTN) clinical trials, HPTN 083 and HPTN 084, but no studies had examined the safety, tolerability, and acceptability in adolescents. Favourable features cited by study participants included its ability to be used discreetly and the infrequent dosing compared with daily tenofovir disoproxil fumarate–emtricitabine PrEP—both attributes that are highly valued by adolescents seeking PrEP for HIV prevention. We searched PubMed using search terms “cabotegravir”, “adolescent”, and “PrEP” for clinical trials published in English between Jan 1, 2018, and Sept 30, 2020, before the initiation of the study, with no results yielded.

**Added value of this study**

This study generated the first data related to long-acting injectable cabotegravir for HIV prevention in adolescent girls. Almost all completed the full course of injections. No scheduled injections were missed, and none were discontinued due to safety events or low tolerability. When exiting the study, most selected long-acting injectable cabotegravir as their HIV prevention method of choice.

**Implications of all the available evidence**

The findings support the licensure of long-acting injectable cabotegravir as HIV PrEP for adolescents. Considering an expanding array of HIV prevention options available for young people, further research is required to optimise PrEP choice and service delivery for young people in need. In parallel to global efforts that promote greater access to new PrEP products, age-specific interventions are necessary for adolescents to navigate the barriers they face with HIV testing and to receive appropriate decisional support for choosing a combination of integrated HIV prevention strategies that suits their individual circumstances.

In adolescents, long-acting formulations of PrEP appear to be particularly well suited for their psychological stage of development and their social environment, including societal stigma related to sexual activity.^5^ Adolescent challenges with adherence and persistence are substantial hurdles to the use of daily oral PrEP, despite multiple mitigation efforts.^6^, ^7^ Younger female participants in early HIV prevention clinical trials and demonstration projects struggled to maintain consistent adherence to oral PrEP.^7^, ^8^, ^9^, ^10^ Overall, longer-acting formulations have the potential to improve PrEP adherence and persistence in this age group, although female adolescents had lower adherence and effectiveness with the monthly dapivirine vaginal ring than older female participants.^11^ Another benefit of long-acting injectable cabotegravir is that there is no associated drug-mediated decline in bone mineral density,^12^ as observed with tenofovir disoproxil fumarate–emtricitabine exposure,^13^, ^14^ which can be important during puberty.

To be licensed for use by adolescent populations for HIV prevention, the safety and pharmacokinetics of long-acting injectable cabotegravir had to be established in this age group. Although substantial differences in the safety profile due to age were unlikely, there were insufficient data from people weighing less than 50 kg. A previous study, IMPAACT 2017 (MOCHA),^15^ evaluated the safety and pharmacokinetics of long-acting injectable cabotegravir among adolescents living with HIV.^15^ However, whether an injection of long-acting injectable cabotegravir every 8 weeks would be acceptable and feasible in the adolescent population for HIV prevention remained to be shown. In this study, HPTN 084-01, we assessed the safety, tolerability, and acceptability of long-acting injectable cabotegravir for PrEP among cisgender female adolescents in three African countries.

## Methods

### Study design and participants

HPTN 084-01 was a single arm, open-label, phase 2b safety, tolerability, and acceptability study conducted among sexually active adolescent girls in three African countries (South Africa, Uganda, and Zimbabwe) with a high burden of HIV. Clinical research sites experienced with conducting research with adolescents affiliated to the HIV Prevention Trials Network (HPTN) implemented the study: Wits Reproductive Health and HIV Institute (Johannesburg, South Africa), Makerere University–Johns Hopkins University Research Collaboration (Kampala, Uganda), and Spilhaus Clinical Research Site (Harare, Zimbabwe). Ethics approval was obtained from research ethics committees and national regulatory authorities in each country. Written assent with informed consent by a parent or legally authorised guardian was obtained in their chosen language from participants for study participation—except for emancipated minors, who provided independent consent—when approved by the institutional review board or independent ethics committee of each clinical research site.

Study participants were healthy, cisgender, adolescent girls recruited by study outreach teams from the community, reproductive health clinics, and through referral by peers or HPTN 084 adult trial participants. Girls were eligible if they were younger than 18 years, weighing at least 35 kg (lowered from initially 50 kg, based on pharmacokinetics data from IMPAACT 2017),^15^ sexually active with male individuals, interested in using long-acting injectable PrEP, and able to involve a parent or legally authorised guardian in the assent or informed consent process. Agreement to use long-acting reversible contraception for the study duration was a prerequisite of participation. Presence of HIV infection, pregnancy, serious medical conditions, harmful substance use, haemoglobin measurements less than 11 g/dL, and substantial renal, hepatic, or cardiovascular disease were exclusionary criteria. Eligibility criteria related to HIV acquisition were expanded after the release of the favourable HPTN 084 clinical trial data.^2^ Subsequently, adolescents who reported recent condomless sexual activity and multiple concurrent sexual partners were eligible for enrolment. HPTN 084-01 is registered with ClinicalTrials.gov (NCT04824131). The full protocol can be found online.^16^

### Procedures

**Figure 1:**
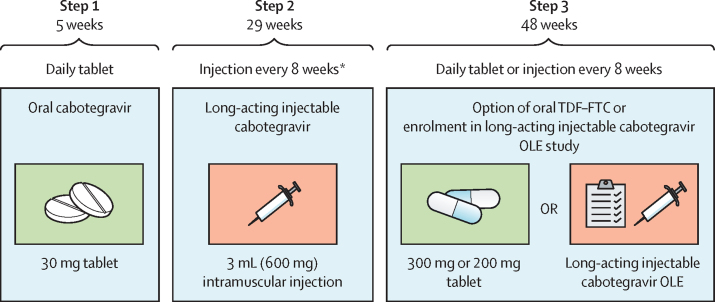
HPTN 084-01 study participation plan Graphics designed by Wits Reproductive Health and HIV Institute (University of the Witwatersrand, Johannesburg, South Africa) and modified by FHI 360 (Durham, NC, USA).OLE=open-label extension. TDF–FTC=tenofovir disoproxil fumarate–emtricitabine. *In step 2, the first two injections are 4 weeks apart and subsequent injections are 8 weeks apart.

The study had three steps (figure 1). Step 1 involved a 5-week lead-in of oral cabotegravir 30 mg taken once daily to confirm tolerability. Clinical and laboratory safety visits were conducted at weeks 2 and 4, when adherence to daily dosing was assessed by tablet counts and supported by counselling.

After confirming tolerability, participants transitioned to step 2, the active injection phase. A series of five intramuscular injections of 3 mL (600 mg) of long-acting injectable cabotegravir were administered by study clinicians into the gluteal muscle via a sterile hypodermic needle, 23G × 25 mm or 38 mm for participants with BMI less than or equal to 30 kg/m^2^ and 23G × 50 mm for BMI more than 30 kg/m^2^. An initial dose was administered at study week 5, with a repeat dose administered 4 weeks later (week 9) and subsequent injections every 8 weeks thereafter (weeks 17, 25, and 33), each visit having a 3-day window before or after a target study visit date. Weight and height were measured at each injection visit. Before each injection, participants had a urine pregnancy test and were tested for HIV with rapid antibody tests and laboratory-based antibody and antigen assays consistent with the strategy used during the blinded phase of HPTN 084. Laboratory screening for other sexually transmitted infections (STIs; cervicovaginal swabs for nucleic acid amplification for *Chlamydia trachomatis* and *Neisseria gonorrhoea*, and serologies for syphilis, hepatitis B, and hepatitis C) was done at baseline, week 33, and if clinically indicated. HIV risk-reduction counselling was provided at each study visit in accordance with local practice. Adherence support, also provided at each study visit, emphasised the importance of returning for injection visits on or as close to the scheduled date as practical.

Clinical and laboratory safety assessments were performed at baseline and 1 week after each injection (weeks 6, 10, 18, 26, and 34) to document injection site reactions (ISRs) and renal, hepatic, and haematological toxicity. Plasma was drawn by study phlebotomists at each scheduled visit and stored in case retrospective testing was required to clarify HIV acquisition. All adverse events and ISRs were assessed and recorded by study clinicians in the study source documents. Severity was assessed by study clinicians with the US National Institute of Allergy and Infectious Diseases (NIAID) Division of AIDS (DAIDS) Table for Grading the Severity of Adult and Pediatric Adverse Events,^17^ and the relatedness of adverse events to long-acting injectable cabotegravir assigned by the site investigator. Adverse events of special interest for long-acting injectable cabotegravir were seizures, neuropsychiatric events, and hepatic toxicity requiring product discontinuation. Depressive symptoms were measured with the Center for Epidemiologic Studies Depression Scale^18^ and post-traumatic stress disorder was measured with the 4-item Primary Care Post-Traumatic Stress Disorder Screen.^19^

Participants transitioned to step 3 for continued observation after discontinuing long-acting injectable cabotegravir injections as scheduled, or prematurely because of becoming pregnant or experiencing predetermined toxicity events (ie, grade >3 adverse event). In step 3, participants were provided with locally sourced, standard-of-care, daily oral tenofovir disoproxil fumarate–emtricitabine as PrEP for the recommended duration of 48 weeks after the last long-acting injectable cabotegravir injection to provide HIV prevention during the tail period of waning concentrations of cabotegravir. When each country team had obtained institutional review board or independent ethics committee approval, active participants who had completed step 2 were offered to continue injectable long-acting injectable cabotegravir in the open-label extension (OLE) phase of the adult HPTN 084 study, the results of which will be reported separately.

Participants reported sexual behaviour through computer-assisted self-interview at baseline, week 4, and at each injection visit. Further, PrEP product satisfaction and acceptability were assessed at baseline and periodically by computer-assisted self-interview, which also reported their attitudes towards study participation, perception of HIV risk, mental health, substance use, and experience of intimate partner violence. Individualised counselling was offered at each scheduled visit to support adherence and promote mental wellbeing. Social harm was assessed at each scheduled visit by study staff who inquired directly whether the participant had experienced any harm since the previous visit.

### Outcomes

The primary outcomes were safety, tolerability, and acceptability. The safety endpoint was the percentage of participants experiencing any grade 2 or higher clinical adverse events and laboratory abnormalities, assessed in all enrolled participants. Tolerability was assessed as the percentage of participants who received at least one injection and who discontinued injections before the full course due to an intolerability to the injection, pain, swelling, or redness, or a disapproval of the frequency of injections or burden of study procedures. Acceptability was measured by the percentage of participants who completed all scheduled injections and the proportion of participants who received at least one injection who would prefer using long-acting injectable cabotegravir over other products for HIV prevention in the future. Separate publications will report the prespecified secondary endpoints of change in sexual behaviour (ie, number of sexual partners and episodes of unprotected anal and vaginal intercourse) during the injection phase, a descriptive analysis of plasma concentration of long-acting injectable cabotegravir, and qualitative data collected via interviews of adolescent participants and parents or guardians.

### Statistical analysis

The target sample size was approximately 50 participants, the number agreed upon by the industry sponsor and regulators to provide sufficient safety and behavioural data on adolescents to supplement the data from HPTN 083 and HPTN 084 adult trials. An additional five participants were enrolled to accommodate potential loss to follow-up. To calculate the final Z scores and establish baseline BMI categories for age, we used the WHO tool.^20^ Standard descriptive summary statistics (ie, counts, proportions, means and SDs, and medians and IQRs) were provided; no inferential testing was performed. We used SAS (version 9.4) for statistical analyses.

To assess safety, the number and the percentage of participants experiencing each safety endpoint were tabulated. Each participant contributed once in each adverse event category according to the highest severity adverse event reported (eg, if one participant had a grade 3 and a grade 4 event in the category, only the grade 4 event was counted for the purposes of analysis). The number and percentage of participants experiencing each type of ISR sign or symptom were tabulated by severity. For a given sign or symptom, each participant's ISR was counted once under the maximum severity for all injection visits and by each successive injection. In addition, the proportion of participants experiencing ISRs and the number of injections that resulted in an ISR were reported. There was a data monitoring committee.

### Role of the funding source

The funders of the study had no role in the study design, data collection, data analysis, data interpretation, or writing of the report, but contributed to the review of the report.

## Results

**Figure 2:**
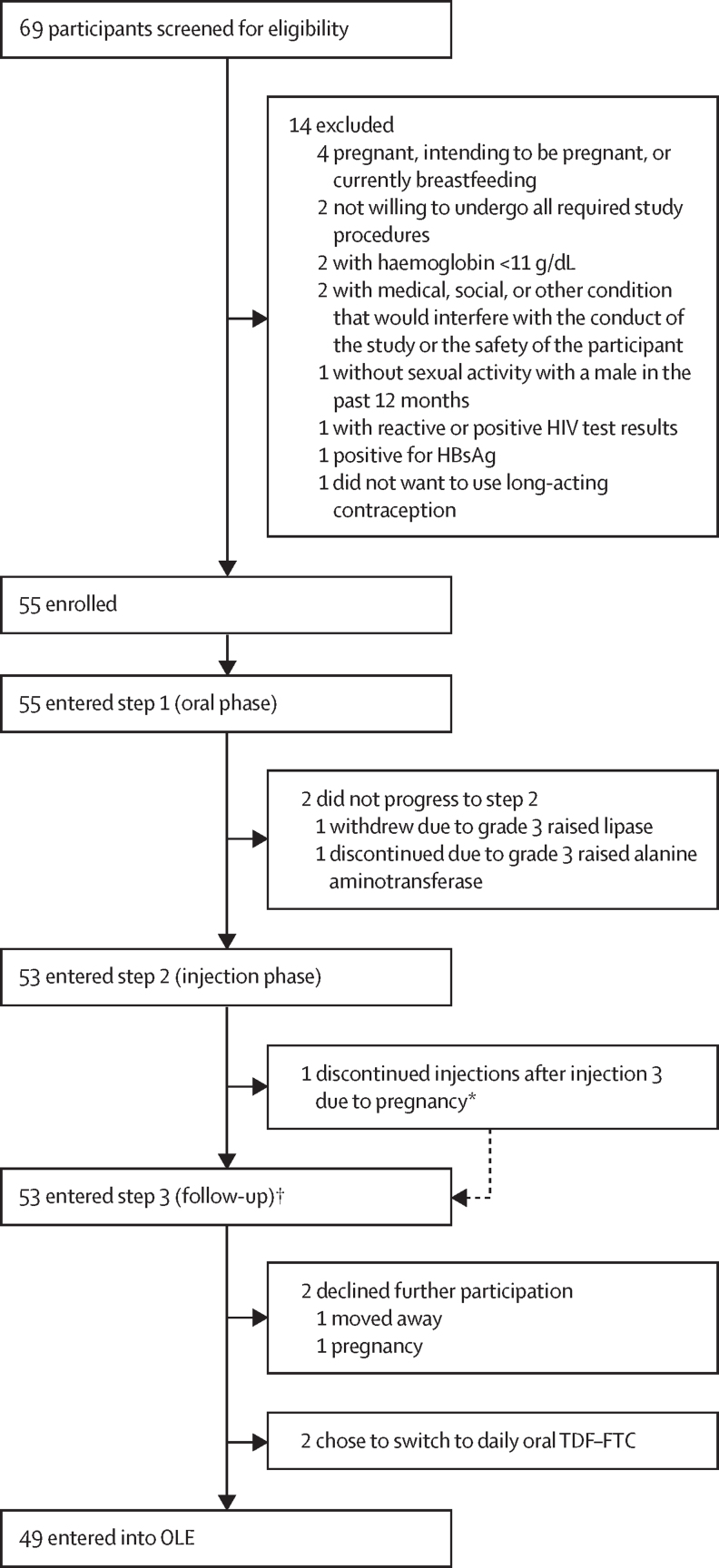
Trial profile OLE=open label extension. TDF–FTC=tenofovir disoproxil fumarate–emtricitabine. *Did not receive injections 4 and 5 but transitioned to OLE after completing a few weeks of follow-up in step 3 before week 8. †48 participants were followed up at week 8, 36 at week 12, 25 at week 24, 15 at week 36, and three at week 48.

Between Nov 1, 2020, and Aug 31, 2021, 69 adolescent girls provided assent with parent or guardian consent and were screened for this study (figure 2), of whom 14 did not meet all eligibility criteria and were excluded. 55 participants were ultimately enrolled and all initiated the oral lead-in phase. Two participants did not transition to step 2 and did not receive any long-acting injectable cabotegravir injections: one participant had elevated lipase at the week 2 visit (grade 3) and refused further participation, withdrawing from the study at week 5, and the second participant was withdrawn as per prespecified criteria after experiencing grade 3 elevated alanine aminotransferase at the week 4 visit. Of the 53 participants who entered step 2, one participant had a positive pregnancy test after three long-acting injectable cabotegravir injections, did not receive the final two injections and instead transitioned to step 3, and was provided with oral tenofovir disoproxil fumarate–emtricitabine. The remaining 52 participants received all five scheduled long-acting cabotegravir injections to complete step 2. No participants were lost to follow-up. No participant acquired HIV infection during steps 1 and 2.

The mean age at study entry was 16·0 years (SD 1·1) and 15 (27%) participants were younger than 16 years. Seven (13%) participants had no regular housing and 31 (56%) were worried about food security (table 1). 20 (36%) participants had depressive symptoms and nine (16%) had potential for post-traumatic stress disorder. 21 (38%) participants reported intimate partner violence occurring in the 6 months before study entry: there were 18 reports of verbal abuse, eight reports of physical abuse, and six reports of sexual abuse (some participants reported more than one type). Problematic alcohol and other substance misuse were reported infrequently. In the month before study entry, 39 (71%) reported having sex, 16 (29%) had two or more male partners, 13 (24%) had a male sexual partner with known HIV infection, and 12 (22%) engaged in transactional sex with a man. The median number of episodes of vaginal sex in the month before study entry was two (IQR 1–4), of which a median of one (0–2) was condomless. Anal sex was reported by three (5%) participants. At baseline, 22 (40%) participants tested positive when screened for STIs; 17 (31%) had cervicovaginal *C trachomatis*, four (7%) had cervicovaginal *N gonorrhoea*, and one (2%) had syphilis. When asked to rate their personal risk of acquiring HIV, 20 (36%) participants reported being not at any risk, ten (18%) reported being a little at risk, and 25 (45%) reported being at a lot of risk.

**Table 1:** Characteristics at study entry (sociodemographic, sexual behaviour, common mental disorders, and substance use)

		**Participants (N=55)**
Weight <50 kg		15 (27%)
Mean BMI age-standardised Z score		0·4 (0·9)
	Obese*	2 (4%)
	Overweight*	14 (25%)
	Typical weight*	38 (69%)
	Thin*	1 (2%)
	Severely thin*	0 (0%)
Housing insecurity		7 (13%)
Food insecurity		31 (56%)
Sexual activity in the previous month		
	Median male sexual partners	1 (1–2)
	Had a primary sexual partner in previous month	39 (71%)
	Primary sexual partner of unknown HIV status	11 (20%)
	Transactional sex	12 (22%)
	Have male sexual partner(s) with known HIV	14 (25%)
	Episodes of vaginal sex	2 (1–4)
	Episodes of vaginal sex without condom	1 (0–2)
	Anal sex	3 (5·5)
Sexually transmitted infection (cervicovaginal)		
	*Chlamydia trachomatis*	17 (31%)
	*Neisseria gonorrhoea*	4 (7%)
	Syphilis	1 (2%)
Intimate partner violence in previous 6 months		
	Physical	8 (15%)
	Verbal	18 (33%)
	Forced sex	6 (11%)
	Felt in danger from male partner	7 (13%)
	Perception of HIV risk as not at all	20 (36%)
	Depressive symptoms†	20 (36%)
	Potential PTSD‡	9 (16%)
Alcohol use		
	Monthly, less, or never	40 (73%)
	Hazardous§	10 (18%)
	Before sex in the previous month	7 (13%)
Cannabis use in the previous month		6 (11%)
Contraceptive method chosen		
	Implant	26 (47%)
	Injectable	29 (53%)

Data are n (%), mean (SD), or median (IQR). PTSD=post-traumatic stress disorder.

* Z scores more than +2 (obesity), more than +1 to less than +2 (overweight), more than −2 to less than +1 (typical weight), more than −3 to less than −2 (thinness), and less than −3 (severe thinness).

† Defined as a score of ≥10 on the 10-item Center of Epidemiologic Studies Depression Scale.

‡ Defined as a score of ≥3 on the Primary Care PTSD Screen for DSM-5.

§ Defined as six or more alcoholic drinks on one occasion monthly or more frequently.

Overall, 484 adverse events of all severity grades were reported by the 55 participants during steps 1 and 2 (appendix p 3). 51 (93%) participants reported at least one adverse event of grade 2 (moderate) or higher (table 2), with the first event occurring during step 1 for 31 (56%) participants. The most frequently recorded grade 2 or higher adverse events were transient asymptomatic laboratory abnormalities, most being unrelated to long-acting injectable cabotegravir (n=51; 41 [75%] of 55 participants had decreased creatinine clearance, 14 had increased amylase, eight had increased serum creatinine, and eight had decreased blood glucose), followed by infectious conditions (n=28; eight had urinary tract and five had upper respiratory tract infections), and reproductive system disorders (n=14; eight had abnormal uterine bleeding and three had intermenstrual bleeding; table 2; appendix p 3 for all events). The alternative cause most commonly provided by the site investigators for decreased creatinine clearance was dehydration. One participant had suicidal ideation after a negative social interaction with a close family member, considered not related to long-acting injectable cabotegravir by study investigators. Apart from that instance, there were no further adverse events of special interest. There were no product-related serious adverse events or injection discontinuations due to adverse events. BMI Z scores did not increase from baseline: two (4%) participants were categorised as obese at study entry and two (4%) at the last injection visit, and 14 (26%) were categorised as overweight at study entry and 12 (23%) at the last injection visit. ISRs were reported by 14 (26%) participants after 15 (6%) of the 263 injections administered, including injection site pain (nine at grade 1 and four at grade 2), tenderness (two at grade 1 and two at grade 2), induration (two at grade 1), and swelling (one at grade 1). ISRs occurred most frequently at the first (n=9) or second (n=4) injection (appendix p 4).

**Table 2:** Number of participants reporting one or more grade 2 and higher adverse events by system organ class and site

	**Overall (N=55)**	**South Africa (N=18)**	**Uganda (N=17)**	**Zimbabwe (N=20)**
Investigations	51 (93%)	16 (89%)	16 (94%)	19 (95%)
Infections and infestations	28 (51%)	8 (44%)	8 (47%)	12 (60%)
Reproductive system and breast disorders	14 (25%)	6 (33%)	3 (18%)	5 (25%)
Gastrointestinal disorders	6 (11%)	5 (28%)	0	1 (5%)
Metabolism and nutrition disorders	5 (9%)	5 (28%)	0	0
Injury, poisoning, and procedural complications	4 (7%)	2 (11%)	2 (12%)	0
Renal and urinary disorders	4 (7%)	1 (6%)	2 (12%)	1 (5%)
Musculoskeletal and connective tissue disorders	3 (5%)	2 (11%)	0	1 (5%)
Skin and subcutaneous tissue disorders	3 (5%)	3 (17%)	0	0
Eye disorders	2 (4%)	2 (11%)	0	0
Nervous system disorders	2 (4%)	2 (11%)	0	0
Psychiatric disorders	2 (4%)	1 (6%)	0	1 (5%)
Blood and lymphatic system disorders	1 (2%)	1 (6%)	0	0
General disorders and administration site conditions	1 (2%)	0	1 (6%)	0

Includes only adverse events of grade 2 and above that have been assigned Medical Dictionary for Regulatory Activities codes by clinical staff. Injection site reactions are not included. A full breakdown of each category is provided in the appendix (pp 3–4).

Oral cabotegravir pill counts indicating less than 75% adherence were observed in four (7%) participants at week 2 and five (9%) participants at week 4 (appendix p 5). No scheduled long-acting injectable cabotegravir injections were missed. Of the 263 injections administered, 228 (87%) were provided in the intended 3-day target window before or after a target study visit date, 31 (12%) were given before the target window opened, and four (2%) were administered after the target window closed. With the wider product-specific target window of 7 days before or after the target study visit date, as recommended by the manufacturer, 11 (4%) were given earlier and one (<1%) was given later than recommended. Long-acting injectable cabotegravir injections were well tolerated overall: regarding the coprimary endpoint of tolerability, no participants discontinued injections before the full course of injections due to an intolerability of injection, the frequency of injections, or the burden of study procedures.

After the third injection, participants were assessed by computer-assisted self-interview regarding perceptions around long-acting injectable cabotegravir injections (table 3). Of the 53 participants who received at least one injection, the most desirable attributes of injections included the protective effect against HIV acquisition (29 [55%]), ease of use (22 [42%]), longer-term protection (12 [23%]), and discretion (ten [19%]). 19 (36%) participants reported no concerns with injections; among those who did report concerns, the most frequently reported concerns were potential pain (15 [28%]), potential side-effects (ten [19%]), and the irreversible nature of injections (seven [13%]). Regarding the acceptability endpoint, of the 52 participants surveyed after receiving the fifth long-acting injectable cabotegravir injection, 32 (62%) reported they would prefer using long-acting injectable cabotegravir over other protection products for HIV prevention in the future (table 3).

**Table 3:** Acceptability and recommendations for improvement of injectable PrEP at the end of study by injection population

			**Overall**
Total participants enrolled			55
Participants who received at least one injection			53 (96%)
Participants who completed the acceptability assessment at the end of step 2 (week 33)			52 (95%)
Preferred product for HIV prevention*			
	None		1/52 (2%)
	Condoms only		7/52 (13%)
	Oral PrEP pills only		1/52 (2%)
	Injectable PrEP only		12/52 (23%)
	Condoms and oral PrEP pills together		8/52 (15%)
	Condoms and injectable PrEP together		20/52 (38%)
	Prefer not to answer		3/52 (6%)
	Participants who received ≥1 injection and preferred injectable PrEP† at the end of step 2		32/52 (62%)
		95% CI (Wilson score method)	48–74
Positive opinions of the injectable method‡§			
	Might protect against HIV		29/53 (55%)
	Easier to use than other methods		22/53 (42%)
	Might provide longer-term protection than other methods		12/53 (23%)
	Can be used discreetly, without a partner's knowledge		10/53 (19%)
	Is administered by a health-care provider		5/53 (9%)
	Does not interrupt sex		5/53 (9%)
	Nothing		4/53 (8%)
Concerns about the injectable method‡§			
	None		19/53 (36%)
	Might be painful		15/53 (28%)
	Might cause harmful side-effects		10/53 (19%)
	Once injected it cannot be reversed immediately		7/53 (13%)
	Might not protect against HIV		6/53 (11%)
	Can be used discreetly, without a partner's knowledge		3/53 (6%)
	Cost might be unaffordable		1/53 (2%)
Recommendations for improvement of the injectable method*¶			
	None		20/52 (38%)
	Increase the duration of protection (ie, make it work for a longer period of time)		18/52 (35%)
	Receive the injection in the arm instead of the buttock		10/52 (19%)
	Receive the injection in the thigh instead of the buttock		2/52 (4%)
	Reduce the volume of injectable		2/52 (4%)

Data are n (%) or 95% CI. PrEP=pre-exposure prophylaxis.

* Of the participants who completed the acceptability assessment at the end of step 2 (week 33).

† Proportion of participants who chose either injectable PrEP or a combination of condoms and injectable PrEP.

‡ Data were captured on the computer-aided self-interview questionnaire at the step 2, week 17 injection visit.

§ Participants could choose more than one answer.

¶ Data were captured on the computer-aided self-interview questionnaire at either the last injection visit (step 2, week 33), or, if needed, at the follow-up phase (step 3, week 12) visit.

At the completion of the injection phase, 36 (69%) participants reported having sex in the previous month, with a median number of sexual partners of 1 (IQR 1–2, range 0–30). When subsequently offered a choice between daily oral tenofovir disoproxil fumarate–emtricitabine through the national PrEP programme and long-acting injectable cabotegravir with continued follow-up in the HPTN 084 OLE, 49 (92%) of 53 participants elected to continue or resume long-acting injectable cabotegravir and provided informed consent or assent to join the HPTN 084 OLE. The participant who paused long-acting injectable cabotegravir due to pregnancy took oral tenofovir disoproxil fumarate–emtricitabine throughout delivery, per protocol, then opted to join HPTN 084 OLE postpartum and resumed long-acting injectable cabotegravir injections. Of the four (8%) participants who did not transition to the OLE, two participants switched to daily oral tenofovir disoproxil fumarate–emtricitabine PrEP by choice and completed 48 weeks of observational follow-up in step 3 before study exit, one participant became pregnant and declined to continue study participation, and one moved out of the study site area, too far away to regularly return for injections despite interest in resuming long-acting injectable cabotegravir through participation in HPTN 084 OLE.

## Discussion

In the first trial of long-acting injectable PrEP among adolescent girls who are clinically vulnerable to HIV acquisition, we found long-acting injectable cabotegravir to be a safe, tolerable, and acceptable method for HIV prevention. Within the context of a relatively short clinical trial and parent or guardian support per protocol, the retention of adolescent girls receiving long-acting injectable cabotegravir was feasible, with most returning on time for all five injections. Having been provided with information about PrEP and decision-making support during trial participation, this cohort of adolescents were subsequently able to select the PrEP option that suited their individual circumstances and preferences at study exit.

The safety data and ISRs recorded among the study participants were similar to previous reports from trials among adult men who have sex with men, transgender women, and cisgender women using long-acting injectable cabotegravir as PrEP.^2^, ^21^, ^22^, ^23^ Although almost all participants had at least one adverse event of grade 2 severity or higher during trial participation, there were no product-related serious adverse events. The reporting of adverse events of particular interest in adolescents, namely neuropsychiatric events such as symptoms of depression and suicidal ideation, aligned with expectations for the age group studied and was no different from that seen in the adult trials. The involvement of a supportive parent or guardian for participants of HPTN 084-01 likely influenced the mental health outcomes observed. There was only one participant who required a pause in long-acting injectable cabotegravir dosing, which was due to the inadequate pharmacokinetic and safety data during pregnancy at the time. Continuing to collect safety data from women using long-acting injectable cabotegravir during pregnancy and lactation remains a priority, with pregnancy outcomes from 351 confirmed pregnancies that occurred during the HPTN OLE suggesting continued long-acting injectable cabotegravir use is safe for both parent and child.^24^

Before this trial, based on research to date, our expectation was that adolescent girls selecting an HIV prevention option might reject the mild-to-moderate pain and tenderness frequently reported with injections. Fear of injection pain as a barrier to adolescent uptake is cited in research among African adolescent girls about their perceptions of long-acting injectable contraception,^25^ and hesitancy for vaccination against the human papilloma virus^26^ and SARS-CoV-2.^27^, ^28^ However, in contrast, enrolled adolescents were able to tolerate relatively high-volume 3 mL gluteal injections during HPTN 084-01. Their experience aligns with more recently released tolerability findings from adolescents living with HIV enrolled in the IMPAACT 2017 trial.^15^, ^29^ In IMPAACT 2017, most adolescents reported that the injection caused no or little pain. Similarly, the high acceptability of long-acting injectable cabotegravir noted in our study is consistent with previous reports of theoretical preference for long-acting methods for HIV prevention, including injectable PrEP, in surveys among adolescent girls and young women across the eastern and southern African region.^5^, ^30^, ^31^, ^32^ Ongoing qualitative analyses from this study will explore the preferences and values of the adolescents and their parents or guardians, and how they perceive the trade-off between favourable and feared attributes of the injections every 8 weeks that meet their needs, preferences, and fit with their lifestyle.

Although the study population we recruited were not specifically selected for their clinical vulnerability to HIV, participants reported behaviours associated with elevated risk. The enrolled study population reflects the sexual risk profile and prevailing rates of food insecurity, intimate partner violence, and depression of young women generally in the eastern and southern African regions. The study findings highlight the need for effective HIV prevention interventions that align with their individual circumstances and preferences. To understand the potential for change in sexual behaviour associated with elevated risk of acquiring HIV and other STIs after the uptake of long-acting injectable PrEP, additional analyses are underway to describe patterns of condom use, multiple concurrent sexual partners, and incident STIs. The use of nucleic acid testing associated with long-acting PrEP administration among eastern and southern African participants is currently being explored in the HPTN 084 unblinded period and OLE, which is inclusive of adolescent girls and young women who transitioned to that study from HPTN 084-01.

The exceptional retention and completion of protocol procedures throughout the study produced robust data for this primary analysis. Another notable strength of our data is that the three participating countries are generally representative of sexually active communities found in eastern and southern Africa; however, further monitoring of acceptability and tolerance remains necessary as long-acting injectable cabotegravir implementation projects are initiated across the region. A limitation of our study is the relatively short period of time for follow-up, thus continued follow-up as part of HPTN 084 OLE will shed light on longer-term acceptability. Parent or guardian informed consent was a requirement for participation, potentially restricting generalisability of our acceptability findings to adolescents that are comfortable involving an adult family member in sexual health decisions. The endorsement from consenting adults of their child's choice to join the HPTN 084-01 study, use long-acting contraception, and initiate injectable PrEP underscores the concern of parents or guardians for the sexual health of young women in high-burden HIV settings.

The HPTN 084-01 findings support the safety, tolerability, and acceptability of long-acting injectable cabotegravir among adolescents in eastern and southern Africa, offering a well tolerated option for this population that is less adherence-dependent than oral PrEP as part of combination prevention.^16^ Our findings suggest that the approach to safety monitoring for adolescents using long-acting injectable cabotegravir as PrEP could follow that developed for adult users. However, of note is the importance of providing tailored counselling and promoting mental wellbeing for adolescent PrEP users to successfully navigate the multiple decisions involved in protecting themselves from HIV acquisition. There is urgency for the long-acting injectable cabotegravir demonstration projects underway in Africa to include adolescents and to monitor uptake and persistence of long-acting injectable PrEP in this population, which remains disproportionately susceptible to HIV acquisition.^1^

### Contributors

### Data sharing

De-identified participant data can be made available after publication of this Article upon request to the HPTN Statistical and Data Management Center (https://www.hptn.org/contact) with approval of a proposal and signed data use agreement.

## Declaration of interests

CM is an employee of ViiV Healthcare. All other authors declare no competing interests.
