# Supplementary material for: Safety, tolerability, and acceptability of long-acting injectable cabotegravir for HIV prevention in cisgender female adolescents (HPTN 084-01): a single-arm, open-label, phase 2b trial
Source: Lancet HIV. 2025 Mar 12;12(4):e252–60. doi: 10.1016/S2352-3018(24)00310-2 (PMC11961543; doi:10.1016/S2352-3018(24)00310-2)
Supplement: Supplementary appendix [file mmc1.pdf]

THE LANCET
HIV

Supplementary appendix

This appendix formed part of the original submission and has been peer reviewed. We post it as supplied by the authors.

Supplement to: Stranix-Chibanda L, hamilton el, Ngo J, et al. Safety, tolerability, and acceptability of long-acting injectable cabotegravir for HIV prevention in cisgender female adolescents (HPTN 084-01): a single-arm, open-label, phase 2b trial. *Lancet HIV* 2025; published online March 12. https://doi.org/10.1016/S2352-3018(24)00310-2.

Supplemental Appendix

Table of Contents

A. HPTN 084-01 Protocol Team 1

B. Supplemental Table 1—Adverse Events (AEs) of all severity experienced during study participation .. 3

C. Supplemental Table 2 — Number of Participants Reporting Grade 2 and Above Adverse Experiences by System Organ Class/Preferred Term by Site 3

D. Supplemental Table 3—Injection Site Reaction (ISR) frequency, timing and severity 5

E. Supplemental Table 4—Injection Site Reaction (ISR) Onset and Resolution, by Site 5

F. Table 5 — Adherence in Oral Phase by Site 5

A. HPTN 084-01 Protocol Team – [HPTN 084-01 | The HIV Prevention Trials Network]

Study Team Members

- Sybil Hosek (Protocol Chair)
- Lynda Stranix-Chibanda (Protocol Co-Chair)
- Adeola Adeyeye (NIAID Medial Officer)
- Hans ML Spiegel (NIAID Medical Officer)
- Bill Kapogiannis (NICHD Medical Officer)
- Aida Asmelesh (Clinical Monitoring Committee)
- Kathleen (Kate) MacQueen (HPTN Ethics Working Group)
- Linda-Gail Bekker (Protocol Team Member)
- Raphael J. Landovitz (Protocol Team Member)
- Irene Rwakazina (Pharmaceutical Affairs Branch)

HPTN Leadership & Operations (LOC)

Study Team:

- erica hamilton
- Scott Rose
- Amber Babinec
- Gabriela Salinas-Jimenez
- Tanette Headen
- Nirupama Sista

Community Team:

- Marcus Bryan
- Rhonda White
- Molly Dyer

Communications Team:

- Laura Long
- Sam Alvarado
- Eric Miller
- Kevin Bokoch

Finance Team:

- Sarah Stone
- Gloria Pherribo
- Priti Patel

HPTN Laboratory Center (LC)

- Mark Marzinke
- Estelle Piwowar-Manning
- Yaw Agyei
- Ethel Weld

HPTN Statistical & Data Management Center (SDMC)

- Julie Ngo
- Heather Noble
- Jean Paul Pease
- Lynda Emel
- James Hughes
- Sahar Zangeneh
- Brett Hanscom
- Jennifer Schille

Study Site Teams

Ward 21 CRS (South Africa):

- Sinead Delaney-Moretlwe
- Carrie-Anne Mathew

- Elizabeth Helena Roos
- Ishana Naidoo

<u>MU-JHU CRS (Uganda):</u>

- Brenda Gati Mirembe
- Clemensia Nakabiito
- Betty Kamira

<u>Spilhaus CRS (Zimbabwe):</u>

- Nyaradzo Mgodi
- Bekezela Siziba
- Eunice Tahuringana

ViiV Healthcare

- Cindy McCoig
- Alex Rinehart

Bill & Melinda Gates Foundation

- Lut Van Damme

B. **Supplemental Table 1—Adverse Events (AEs) of all severity experienced during study participation**

Overall, 484 AEs were reported for 55 participants enrolled.

Overall						
	Relationship to Study Product					
	Related		Not Related		Total [2]	
	N	%	N	%	N	%
**Severity Grade**						
Mild	64	(27.8%)	166	(72.2%)	230	(47.5%)
Moderate	50	(20.5%)	194	(79.5%)	244	(50.4%)
Severe	0	(0%)	9	(100%)	9	(1.9%)
Potentially Life Threatening	0	(0%)	1	(100%)	1	(0.2%)
Death	0	–	0	–	0	(0%)
Missing	0	–	0	–	0	(0%)
**Total [2]**	114	(23.6%)	370	(76.5%)	484	(100%)

C. **Supplemental Table 2 — Number of Participants Reporting Grade 2 and Above Adverse Experiences [1] by System Organ Class/Preferred Term by Site**

	Overall	MU−JHU	Spilhaus	Ward 21
**Total Participants Enrolled**	**55**	**17**	**20**	**18**
Number of Participants with AEs[2]	51/55 (92.7%)	16/17 (94.1%)	19/20 (95.0%)	16/18 (88.9%)
**Blood and lymphatic system disorders**	**1/55 (1.8%)**	**0/17 (0.0%)**	**0/20 (0.0%)**	**1/18 (5.6%)**
Iron deficiency anaemia	1/55 (1.8%)	0/17 (0.0%)	0/20 (0.0%)	1/18 (5.6%)
**Eye disorders**	**2/55 (3.6%)**	**0/17 (0.0%)**	**0/20 (0.0%)**	**2/18 (11.1%)**
Refraction disorder	1/55 (1.8%)	0/17 (0.0%)	0/20 (0.0%)	1/18 (5.6%)
Uveitis	1/55 (1.8%)	0/17 (0.0%)	0/20 (0.0%)	1/18 (5.6%)
**Gastrointestinal disorders**	**6/55 (10.9%)**	**0/17 (0.0%)**	**1/20 (5.0%)**	**5/18 (27.8%)**
Abdominal pain	1/55 (1.8%)	0/17 (0.0%)	0/20 (0.0%)	1/18 (5.6%)
Aphthous ulcer	1/55 (1.8%)	0/17 (0.0%)	1/20 (5.0%)	0/18 (0.0%)
Constipation	3/55 (5.5%)	0/17 (0.0%)	0/20 (0.0%)	3/18 (16.7%)
Dental caries	1/55 (1.8%)	0/17 (0.0%)	0/20 (0.0%)	1/18 (5.6%)
**General disorders and administration site conditions**	**1/55 (1.8%)**	**1/17 (5.9%)**	**0/20 (0.0%)**	**0/18 (0.0%)**
Pyrexia	1/55 (1.8%)	1/17 (5.9%)	0/20 (0.0%)	0/18 (0.0%)
**Infections and infestations**	**28/55 (50.9%)**	**8/17 (47.1%)**	**12/20 (60.0%)**	**8/18 (44.4%)**
Bacterial vaginosis	2/55 (3.6%)	0/17 (0.0%)	2/20 (10.0%)	0/18 (0.0%)
Cellulitis	2/55 (3.6%)	0/17 (0.0%)	0/20 (0.0%)	2/18 (11.1%)
Chlamydial infection	3/55 (5.5%)	1/17 (5.9%)	0/20 (0.0%)	2/18 (11.1%)
Conjunctivitis bacterial	1/55 (1.8%)	0/17 (0.0%)	0/20 (0.0%)	1/18 (5.6%)
Genital herpes	1/55 (1.8%)	0/17 (0.0%)	1/20 (5.0%)	0/18 (0.0%)
Genitourinary chlamydia infection	3/55 (5.5%)	0/17 (0.0%)	3/20 (15.0%)	0/18 (0.0%)
Genitourinary tract gonococcal infection	3/55 (5.5%)	2/17 (11.8%)	1/20 (5.0%)	0/18 (0.0%)
Gonococcal infection	1/55 (1.8%)	0/17 (0.0%)	0/20 (0.0%)	1/18 (5.6%)
Influenza	2/55 (3.6%)	0/17 (0.0%)	2/20 (10.0%)	0/18 (0.0%)
Malaria	1/55 (1.8%)	1/17 (5.9%)	0/20 (0.0%)	0/18 (0.0%)
Oral herpes	1/55 (1.8%)	0/17 (0.0%)	1/20 (5.0%)	0/18 (0.0%)
Otitis media	1/55 (1.8%)	0/17 (0.0%)	0/20 (0.0%)	1/18 (5.6%)
Respiratory tract infection	1/55 (1.8%)	0/17 (0.0%)	1/20 (5.0%)	0/18 (0.0%)
Tonsillitis	1/55 (1.8%)	0/17 (0.0%)	1/20 (5.0%)	0/18 (0.0%)

Typhoid fever	1/55 (1.8%)	0/17 (0.0%)	1/20 (5.0%)	0/18 (0.0%)
Upper respiratory tract infection	5/55 (9.1%)	3/17 (17.6%)	1/20 (5.0%)	1/18 (5.6%)
Urinary tract infection	8/55 (14.5%)	2/17 (11.8%)	1/20 (5.0%)	5/18 (27.8%)
Vulvovaginitis trichomonal	1/55 (1.8%)	0/17 (0.0%)	1/20 (5.0%)	0/18 (0.0%)
**Injury, poisoning and procedural complications**	**4/55 (7.3%)**	**2/17 (11.8%)**	**0/20 (0.0%)**	**2/18 (11.1%)**
Skin laceration	2/55 (3.6%)	0/17 (0.0%)	0/20 (0.0%)	2/18 (11.1%)
Soft tissue injury	1/55 (1.8%)	1/17 (5.9%)	0/20 (0.0%)	0/18 (0.0%)
Thermal burn	1/55 (1.8%)	1/17 (5.9%)	0/20 (0.0%)	0/18 (0.0%)
**Investigations**	**51/55 (92.7%)**	**16/17 (94.1%)**	**19/20 (95.0%)**	**16/18 (88.9%)**
Amylase increased	14/55 (25.5%)	1/17 (5.9%)	4/20 (20.0%)	9/18 (50.0%)
Blood alkaline phosphatase increased	2/55 (3.6%)	2/17 (11.8%)	0/20 (0.0%)	0/18 (0.0%)
Blood bilirubin increased	4/55 (7.3%)	4/17 (23.5%)	0/20 (0.0%)	0/18 (0.0%)
Blood calcium decreased	1/55 (1.8%)	1/17 (5.9%)	0/20 (0.0%)	0/18 (0.0%)
Blood cholesterol increased	1/55 (1.8%)	1/17 (5.9%)	0/20 (0.0%)	0/18 (0.0%)
Blood creatine phosphokinase increased	2/55 (3.6%)	0/17 (0.0%)	1/20 (5.0%)	1/18 (5.6%)
Blood creatinine increased	8/55 (14.5%)	1/17 (5.9%)	4/20 (20.0%)	3/18 (16.7%)
Blood glucose decreased	8/55 (14.5%)	0/17 (0.0%)	3/20 (15.0%)	5/18 (27.8%)
Blood glucose increased	1/55 (1.8%)	1/17 (5.9%)	0/20 (0.0%)	0/18 (0.0%)
Creatinine renal clearance decreased	41/55 (74.5%)	12/17 (70.6%)	18/20 (90.0%)	11/18 (61.1%)
Lipase increased	6/55 (10.9%)	2/17 (11.8%)	0/20 (0.0%)	4/18 (22.2%)
Low density lipoprotein decreased	1/55 (1.8%)	0/17 (0.0%)	1/20 (5.0%)	0/18 (0.0%)
Low density lipoprotein increased	1/55 (1.8%)	1/17 (5.9%)	0/20 (0.0%)	0/18 (0.0%)
Lymphocyte count decreased	1/55 (1.8%)	0/17 (0.0%)	1/20 (5.0%)	0/18 (0.0%)
Neutrophil count decreased	2/55 (3.6%)	1/17 (5.9%)	1/20 (5.0%)	0/18 (0.0%)
Platelet count decreased	2/55 (3.6%)	2/17 (11.8%)	0/20 (0.0%)	0/18 (0.0%)
**Metabolism and nutrition disorders**	**5/55 (9.1%)**	**0/17 (0.0%)**	**0/20 (0.0%)**	**5/18 (27.8%)**
Abnormal loss of weight	4/55 (7.3%)	0/17 (0.0%)	0/20 (0.0%)	4/18 (22.2%)
Decreased appetite	1/55 (1.8%)	0/17 (0.0%)	0/20 (0.0%)	1/18 (5.6%)
Iron deficiency	1/55 (1.8%)	0/17 (0.0%)	0/20 (0.0%)	1/18 (5.6%)
**Musculoskeletal and connective tissue disorders**	**3/55 (5.5%)**	**0/17 (0.0%)**	**1/20 (5.0%)**	**2/18 (11.1%)**
Arthralgia	1/55 (1.8%)	0/17 (0.0%)	1/20 (5.0%)	0/18 (0.0%)
Costochondritis	1/55 (1.8%)	0/17 (0.0%)	0/20 (0.0%)	1/18 (5.6%)
Myalgia	1/55 (1.8%)	0/17 (0.0%)	0/20 (0.0%)	1/18 (5.6%)
**Nervous system disorders**	**2/55 (3.6%)**	**0/17 (0.0%)**	**0/20 (0.0%)**	**2/18 (11.1%)**
Headache	2/55 (3.6%)	0/17 (0.0%)	0/20 (0.0%)	2/18 (11.1%)
**Psychiatric disorders**	**2/55 (3.6%)**	**0/17 (0.0%)**	**1/20 (5.0%)**	**1/18 (5.6%)**
Stress	1/55 (1.8%)	0/17 (0.0%)	0/20 (0.0%)	1/18 (5.6%)
Suicide attempt	1/55 (1.8%)	0/17 (0.0%)	1/20 (5.0%)	0/18 (0.0%)
**Renal and urinary disorders**	**4/55 (7.3%)**	**2/17 (11.8%)**	**1/20 (5.0%)**	**1/18 (5.6%)**
Proteinuria	4/55 (7.3%)	2/17 (11.8%)	1/20 (5.0%)	1/18 (5.6%)
**Reproductive system and breast disorders**	**14/55 (25.5%)**	**3/17 (17.6%)**	**5/20 (25.0%)**	**6/18 (33.3%)**
Abnormal uterine bleeding	8/55 (14.5%)	0/17 (0.0%)	2/20 (10.0%)	6/18 (33.3%)
Heavy menstrual bleeding	1/55 (1.8%)	1/17 (5.9%)	0/20 (0.0%)	0/18 (0.0%)
Intermenstrual bleeding	3/55 (5.5%)	2/17 (11.8%)	1/20 (5.0%)	0/18 (0.0%)
Ovarian cyst	1/55 (1.8%)	0/17 (0.0%)	1/20 (5.0%)	0/18 (0.0%)
Vaginal discharge	1/55 (1.8%)	0/17 (0.0%)	1/20 (5.0%)	0/18 (0.0%)
**Skin and subcutaneous tissue disorders**	**3/55 (5.5%)**	**0/17 (0.0%)**	**0/20 (0.0%)**	**3/18 (16.7%)**
Dermatitis allergic	2/55 (3.6%)	0/17 (0.0%)	0/20 (0.0%)	2/18 (11.1%)
Livedo reticularis	1/55 (1.8%)	0/17 (0.0%)	0/20 (0.0%)	1/18 (5.6%)

[1] This table includes only those AEs which have been assigned MedDRA codes by clinical staff. Injection Site Reactions are not included.

[2] For participants reporting multiple events with the same MedDRA term, only one is counted. Percentages are calculated as the number of participants (n) reporting an event divided by the number enrolled.

D. Supplemental Table 3—Injection Site Reaction (ISR) frequency, timing and severity

Overall, a total of 6 ISRs of any grade severity were reported. One participant reported 2 separate ISRs of grade 2 or more severity at injections 2 and 4.

	**Injection 1** (n=53)	**Injection 2** (n=53)	**Injection 3** (n=53)	**Injection 4** (n=52)*	**Injection 5** (n=52)*
Any ISR	9	4	1	1	0
Grade 2 or more severity	0	2	1	1	0

* One participant fell pregnant and switched to oral FTC/TDF PrEP until postpartum

E. Supplemental Table 4—Injection Site Reaction (ISR) Onset and Resolution, by Site

	**Overall**	**South Africa**	**Uganda**	**Zimbabwe**
All visits combined				
Total number of injections with Grade 2 and above ISRs	6	0	5	1
Onset same day as injection	3/6 (50.0%)	0/0 (−%)	3/5 (60.0%)	0/1 (0.0%)
Onset 1−2 days after injection	1/6 (16.7%)	0/0 (−%)	0/5 (0.0%)	1/1 (100.0%)
Onset 3−7 days after injection	2/6 (33.3%)	0/0 (−%)	2/5 (40.0%)	0/1 (0.0%)
Onset more than 7 days after injection	0/6 (0.0%)	0/0 (−%)	0/5 (0.0%)	0/1 (0.0%)
ISR resolved on the same day as ISR onset	0/6 (0.0%)	0/0 (−%)	0/5 (0.0%)	0/1 (0.0%)
ISR resolved from 1−2 days from ISR onset	0/6 (0.0%)	0/0 (−%)	0/5 (0.0%)	0/1 (0.0%)
ISR resolved from 3−7 days from ISR onset	1/6 (16.7%)	0/0 (−%)	0/5 (0.0%)	1/1 (100.0%)
ISR resolved after 7 days from ISR onset	5/6 (83.3%)	0/0 (−%)	5/5 (100.0%)	0/1 (0.0%)
ISR Not resolved	0/6 (0.0%)	0/0 (−%)	0/5 (0.0%)	0/1 (0.0%)

F. Table 5 — Adherence in Oral Phase by Site[1]

	**Overall**	**South Africa**	**Uganda**	**Zimbabwe**
Total Participants Enrolled	55	18	17	20
Week 2 CAB Adherence:				
100%	12/55 (22%)	3/18 (17%)	5/17 (29%)	4/20 (20%)
90% to 99%	27/55 (49%)	6/18 (33%)	7/17 (41%)	14/20 (70%)
80% to 89%	12/55 (22%)	5/18 (28%)	5/17 (29%)	2/20 (10%)
75% to 79%	0/55 (0%)	0/18 (0%)	0/17 (0%)	0/20 (0%)
<75%	4/55 (7%)	4/18 (22%)	0/17 (0%)	0/20 (0%)
Week 4 CAB Adherence:				
100%	6/54 (11%)	3/17 (18%)	2/17 (12%)	1/20 (5%)
90% to 99%	29/54 (54%)	6/17 (35%)	9/17 (53%)	14/20 (70%)
80% to 89%	13/54 (24%)	5/17 (29%)	3/17 (18%)	5/20 (25%)
75% to 79%	1/54 (2%)	1/17 (6%)	0/17 (0%)	0/20 (0%)
<75%	5/54 (9%)	2/17 (12%)	3/17 (18%)	0/20 (0%)

1 Data presented are based on pill counts (pill dispensed at previous visit − pill count at current visit)/days between visits.
CAB – Cabotegravir tablets
